# An intragenic approach to confer glyphosate resistance in chile (*Capsicum annuum*) by introducing an *in vitro* mutagenized chile *EPSPS* gene encoding for a glyphosate resistant EPSPS protein

**DOI:** 10.1371/journal.pone.0194666

**Published:** 2018-04-12

**Authors:** Jose Luis Ortega, Wathsala Rajapakse, Suman Bagga, Kimberly Apodaca, Yvonne Lucero, Champa Sengupta-Gopalan

**Affiliations:** Department of Plant and Environmental Sciences, New Mexico State University, Las Cruces, New Mexico, United States of America; USDA/ARS, UNITED STATES

## Abstract

Chile pepper (*Capsicum annuum*) is an important high valued crop worldwide, and when grown on a large scale has problems with weeds. One important herbicide used is glyphosate. Glyphosate inactivates the enzyme 5-enolpyruvylshikimate-3-phosphate synthase (*EPSPS*), a key enzyme in the synthesis of aromatic amino acids. A transgenic approach towards making glyphosate resistant plants, entails introducing copies of a gene encoding for glyphosate-resistant EPSPS enzyme into the plant. The main objective of our work was to use an intragenic approach to confer resistance to glyphosate in chile which would require using only chile genes for transformation including the selectable marker. Tobacco was used as the transgenic system to identify different gene constructs that would allow for the development of the intragenic system for chile, since chile transformation is inefficient. An *EPSPS* gene was isolated from chile and mutagenized to introduce substitutions that are known to make the encoded enzyme resistant to glyphosate. The promoter for *EPSPS* gene was isolated from chile and the mutagenized chile *EPSPS* cDNA was engineered behind both the *CaMV35S* promoter and the *EPSPS* promoter. The leaves from the transformants were checked for resistance to glyphosate using a cut leaf assay. In tobacco, though both gene constructs exhibited some degree of resistance to glyphosate, the construct with the *CaMV35S* promoter was more effective and as such chile was transformed with this gene construct. The chile transformants showed resistance to low concentrations of glyphosate. Furthermore, preliminary studies showed that the mutated *EPSPS* gene driven by the *CaMV35S* promoter could be used as a selectable marker for transformation. We have shown that an intragenic approach can be used to confer glyphosate-resistance in chile. However, we need a stronger chile promoter and a mutated chile gene that encodes for a more glyphosate resistant EPSPS protein.

## Introduction

Chile pepper (*Capsicum annuum*) is an important high valued crop worldwide. Besides all its nutritional properties, chile pepper has some unique features such as its ability to synthesize different kinds of pigments that are used as non-toxic coloring compounds, and the capsaicinoids that have therapeutic properties [1–3]. Chile like many other crops grown on a large scale has the usual problems with weeds.

One of the important herbicide used is glyphosate and it is used in chile fields to a limited extent. Glyphosate resistant crops like soybean, cotton, alfalfa, sugar beet and corn have been rapidly adopted because use of glyphosate is a superior and more environmentally friendly weed control method [4, 5]. Glyphosate inactivates the enzyme 5-enolpyruvylshikimate-3-phosphate synthase (EPSPS). EPSPS is a key enzyme in the synthesis of aromatic amino acids, and catalyzes the transfer of the enolpyruvyl moiety of phosphoenolpyruvate (PEP) to the 5’ hydroxyl position of shikimate-3-phosphate (S3P). Binding of S3P to the enzyme triggers a global conformational change from an “open” to a “closed” conformation [6]. Inhibition of EPSPS by glyphosate appears to be competitive with respect to PEP and it has been shown that glyphosate occupies the PEP-binding site [6, 7]. Thus glyphosate, by inactivating EPSPS, blocks the synthesis of aromatic amino acids, leading to plant death.

To be able to use glyphosate in crop fields, glyphosate resistant plants have been created by over expressing bacterial *EPSPS* genes [8–11]. Among these, the *EPSPS* gene from *Agrobacterium tumefaciens* CP4 has been used the most to generate available commercial glyphosate-resistant crop plants [12, 13]. A gene for a highly glyphosate-resistant EPSPS protein has also been isolated from *Pseudomonas fluorescences* strain G2. This gene conferred resistance to high concentrations of glyphosate in tobacco, cotton, maize and rice [14–17]. More recently, however, people have become more concerned about using bacterial genes in food crops. The alternative approach has been to use plant genes that encode for glyphosate resistant EPSPS [18]. The glyphosate insensitivity of EPSPS is a result of either natural mutations or directed *in vitro* modifications at the glyphosate-binding site of the enzyme [19, 20]. *In vitro* mutated plant *EPSPS* genes have also been used to confer resistance to glyphosate in different crop plants [18, 20]. Tian et al. [21, 22] have used *in vitro*-directed shuffling of the *EPSPS* genes from *Malus domestica* and *Vitis vinefera* to produce glyphosate-resistant EPSPS.

The main deterrent in developing glyphosate resistant EPSPS variants through mutagenesis is that glyphosate resistance is frequently associated with a decrease in the enzyme’s affinity for PEP, which eventually reduces the enzyme’s catalytic efficiency. For example, EPSPS variants carrying single amino acid mutations at glycine 101 or threonine 102 endow high level of glyphosate resistance but with drastically reduced catalytic activity [23]. Since glyphosate binds to the same site where PEP binds, any alterations that disturb glyphosate binding can also affect PEP binding. Hence, in producing glyphosate resistant EPSPS variants, caution has to be taken to replace the amino acid/s that affect glyphosate binding with little to no effect on the binding of PEP. Along with the single site mutations, double site mutations (Threonine 102 to Isoleucine and Proline106 to Serine, TIPS), as identified in *A*. *tumefaciens* CP4 have been made in the maize *EPSPS* gene and successfully used to develop glyphosate resistant transgenic corn [18]. A naturally evolved TIPS mutation has now been found in *Eleusine indica* and this TIPS mutant is highly resistant to glyphosate compared to the wild type [23].

While the use of transgenic crops is on the rise, the gene modification (GM) technology is still being met with substantial skepticism among the general public and the growers. One of the major concerns of the public about transgenic crops is the artificial combination of elements derived from different organisms that cannot be obtained by natural means [24]. Plant transformation requires the use of a selectable marker and bacterial genes have been routinely used for selecting transformed plants [25], and the use of a selectable marker derived from bacterial source is also of great concern among the anti-GM groups.

As an alternative to plant transformation involving transfer of genes from sources other than a crossable plant, we could use a cisgenic and/or intragenic approach which involves the use of genetic material from closely related species that are capable of sexual crosses. In cisgenesis, the origin of the gene for transfer is an identical copy of the endogenous gene including the promoter, introns and the terminator. The intragenes on the other hand, can have different elements of different genes from the same crossable plant. Furthermore, foreign sequences such as selectable markers should also be absent. The cisgenic approach has been used to produce apple resistant to scab [26] and to fire blight [27]. Cisgenic barley has also been produced with improved phytase activity [28]. In all these studies, recombination mediated elimination of the selectable marker gene was used.

Our goal was to produce glyphosate resistant chile plants using an intragenic approach. Though solanaceous plants are fairly easy to transform, chile is fairly recalcitrant to transformation. As such our first challenge was to develop a transformation system for chile plants using the *Agrobacterium tumefaciens* mediated protocol [29]. Using the protocol that we have developed we introduced a chile *EPSPS* gene into chile.

We isolated a cDNA for the *EPSPS* gene from chile (*CaEPSPS*) and based on the information obtained from the literature, introduced mutations at key sites in the gene, with the goal of making a glyphosate insensitive enzyme [23]. To produce an intragene, we also isolated the *EPSPS* promoter from chile. Gene constructs with the modified *CaEPSPS* gene driven by either the *CaMV35S* promoter or the chile *EPSPS* promoter, were introduced into tobacco to test for their efficacy in conferring glyphosate resistance. The gene construct identified to be the most effective construct was introduced into chile. These chile transformants exhibited resistance to moderate levels of glyphosate. Moreover, we have also tested the feasibility of using this gene construct as a selectable marker.

## Materials and methods

### Isolation of the *EPSPS* cDNA and *EPSPS* promoter from chile

The *Capsicum annuum* 5-enolpyruvylshikimate-3-phosphate synthase (*CaEPSPS*) cDNA was cloned from chile leaves by RT-PCR amplification. RNA was reversed transcribed with an oligo dT containing the anchored primer 5’-CCAGTGAGCAGAGTGACGAGGT(17)-3’. PCR amplification was carried out with an *EPSPS* specific primer from a conserved region of a reported tomato *EPSPS* cDNA (GeneBank accession no. NM_001308947.1; 5’-GTG GTT GGT GAA GCT GAC CAT T-3’) and the anchored primer at the 3’ end of the cDNA. A *CaEPSPS* cDNA lacking the 5’UTR and the region encoding the first five amino acids was cloned with this strategy (GeneBank accession no. JN160845.1). A full length *CaEPSPS* cDNA was isolated from chile leaves cv. NM64, by 5’ RACE. RNA was dephosphorylated, 5’ RNA cap was removed and a 25 nucleotides long RNA adapter was ligated to the 5’ ends of mRNAs. *CaEPSPS*-specific cDNA was synthesized using a primer in the 3’UTR of the *CaEPSPS* mRNA (5’-CTT GGA TCA AAG CTT TTC CTG-3’). The complete coding region, including the 5’UTR, was amplified by PCR with primers for the RNA adapter and a primer at the end of the coding region that included a *BstEII* site for further cloning (5’-GGT CAC CTT AGT GCT TGG AGT ACT GCT GG-3’). A second *BstEII* site was introduced at the beginning of the cDNA by PCR amplification.

A genomic DNA fragment containing the chile *EPSPS* promoter was amplified with the Genome Walker kit (Clonetech Laboratories), using primers in the *CaEPSPS* coding region (5’-AGG GCA GCA AGA AGG AGA ACT CGA TTG GAA AGT GAC-3’ and 5’-CTC ATT GGG TGC CTC TGC AGT AGC CAC TGA TG-3’). A promoter fragment comprising 1437 bp upstream of the start codon was cloned in the pBluescript II KS (+) vector.

### Bioinformatic analysis

*EPSPS* nucleotide sequences were retrieved from the NCBI (National Center for Biotechnology Information) database by BLAST search using the *Capsicum annuum EPSPS* cDNA as query. Sequence alignments were carried out using Geneious 9.1 (Biomatters, Ltd). The phylogenetic analysis of *EPSPS* sequences was performed by the neighbor-joining building method with bootstrap values that correspond to 1000 replicates.

### Cloning and construction of the gene cassettes for plant transformation

Two mutations were introduced in the *CaEPSPS* cDNA by PCR-based site-directed mutagenesis to simultaneously change the amino acids Thr179 to Ile and Pro183 to Ser (TIPS). These sites were selected based on the glyphosate insensitive *Zea mays* EPSPS mutant T102I/P106S [18]. A pBluescript II KS (+) plasmid containing the *CaEPSPS* cDNA was ampified by PCR with the primers 5’-TTC CTG CAT TTC CAA GCA ACA GTT GGA TTT CTT C-3’ (reverse) and 5’-GAA ATG CAG GAA TAG CGA TGC GGAGCT TGA CAG CAG-3’ (forward, with the nucleotide changes underlined). The mutagenized plasmid was digested with *DpnI* and transformed into *E*. *coli*.

To produce *CaMV35S*::*CaEPSPS* gene constructs for plant transformation, an intermediate cloning vector was made by inserting the *CaMV35S* promoter and the *A*. *tumefaciens NOS* 3’UTR in the pBluescript II KS(+) plasmid, both linked by a *BstEII* restriction site. The *CaMV35S* promoter was amplified by PCR from the binary plasmid *pCAMBIA2301*, adding *HindIII* and *BstEII* sites to the 5’ and 3’ ends, respectively. The *NOS* terminator, with *BstEII* and *PstI* restriction sites at the 5’ and 3’ ends, was amplified by PCR from pMON316 binary plasmid. The unchanged and the mutagenized *CaEPSPS* cDNAs were inserted as *BstEII* fragments in between the *CaMV35S* promoter and the *NOS* terminator of the intermediate vector.

To make the *EPSPS* promoter::*CaEPSPS* gene construct, a *SalI / EcoRI* region that included the *CaMV35S* promoter and 483 bp of the coding region was removed from the pBluescript /*CaMV35S*::*CaEPSPS* intermediate plasmids and replaced with a *SalI* / *EcoRI* fragment that contained the *EPSPS* promoter region 1437 bp upstream of the start codon and 483 bp of the coding region. The *CaMV35S* promoter and *EPSPS* promoter gene constructs were then released as *KpnI / BamHI* fragments, and cloned in the *pCAMBIA2300* binary plasmid for plant transformation.

### Purification of EPSPS recombinant protein and production of antibodies

A *CaEPSPS* cDNA without the region coding for the transit peptide was amplified by PCR by using a forward primer 5’GGATCCGCAGAGGCACCCAATGAGATTGTG-3’ and the reverse primer at the end of the coding region. The putative transit peptide cleavage site was predicted by comparing the amino acid sequence to the *Z*. *mays* and the *Petunia hybrida* EPSPS sequences (GeneBank Accession nos. X63374.1 and AAA33699.1). The *CaEPSPS* cDNA encoding the mature protein was cloned as a Glutathione Transferase (GST) fusion in the expression vector pGEX-4T-2 (GE Healthcare Life Sciences, Pittsburgh, PA). The EPSPS recombinant protein expressed in *E*. *coli* was purified by chromatography in a Sepharose-GST column, followed by ion exchange chromatography in DEAE-Sephacel. The GST tag was removed by thrombin digestion and an additional Sepharose-GST chromatography. The purified protein was sent out for antibody production to Robert Sargeant (Ramona, CA).

### Plant transformation and analysis of transgenic plants

Tobacco transformation with the different gene constructs was carried out following standard procedures [30]. Chile transformation was carried out using a modified version of the protocol by Liu et al. [31], as described in Rajapakse, 2015 [29]. Cotyledons with attached petioles were cut from 10 to 12 days old chile seedling grown aseptically and preconditioned for 2 days prior to transformation. The *Agrobacterium* inoculated explants were co-cultured for two days and then moved to media for growth and development. Shoot formation at the end of the petioles was observed in 3 to 4 weeks. Individual shoots were cultured on media for root initiation. The transgenic shoots were rooted on antibiotic selection, transferred to soil and moved to the green house for seed setting. All cultures were placed in an incubator with a regimen of 16 h in light and 8 h in the dark at 26°C.

To check for the insertion of the transgene and its expression, genomic PCR and RT PCR was done using *NPTII* specific primer sets. Western blot analysis was done using chile EPSPS antibodies.

### Protein extraction and western blot analysis

Leaf tissue was ground in liquid N with 20% (w/w) insoluble polyvinylpolypyrrolidone and homogenized with 12.5 volumes of ice-cold extraction buffer containing 50 mM Tris-Cl, (pH 8.0), 1 mM EDTA, 1mM magnesium acetate, 5% (v/v) ethylene glycol, 20% (v/v) glycerol, 10 mM DTT and a protease inhibitor cocktail (Thermo Scientific, Rockford, IL). The homogenate was centrifuged and the supernatant was used for western blot analysis. 5 μg of total protein was fractionated on 12% polyacrylamide gel (Mini-PROTEAN TGX Gels, Bio-Rad, Hercules, CA), blotted onto Immobilon-P PVDF Transfer Membrane (EMD Millipore, Billerica, MA) and incubated with rabbit anti-chile EPSPS antibodies.

### RNA extraction and quantitative RT-PCR

Total RNA was extracted from the leaves of transgenic and non-transformed tobacco plants using RNeasy Plant Mini Kit (QIAGEN, Valencia, CA). The RNA quality and concentration were measured using a NanoPhotometer (Implen, Munich, Germany). The cDNA was synthesized from 2 μg of total RNA using SuperScript VILO MasterMix (Invitrogen, Carlsbad, CA) according to the manufacture’s procedure. 10 ng of cDNA was used for quantitative real-time PCR (qRT-PCR) with SsoAdvanced Universal SYBRGreen Supermix (Bio-Rad, Hercules, CA) in a Bio-Rad CFX Connect Real-Time System. The primers were designed using PrimerQuest Tool (Integrated DNA Technologies, Inc). *CaEPSPS*-specific primers (forward: 5’-CTG AAG TTG ATG GAG CGA TTT G-3’ and reverse: 5’-CTT TCC CAG GAG ACT TGT ACT T-3’) were used at 375 nM concentration in qRT-PCR with the following cycling parameters: 3 min at 95°C and 39 cycles of 10 sec at 95°C, 30 sec at 58°C and 72°C for 30 sec. *Nicotiana tabacum* elongation factor 1α (GeneBank accession no. AF120093) was used as the internal reference gene to normalize EPSPS gene expression [32]. The specificity of the primer pairs was verified by melting curve analysis (65–95°C). Prior to qRT-PCR the primers were tested by PCR followed by agarose gel electrophoresis to confirm the amplification of a single product of the expected size. Amplification efficiency and correlation coefficient for all primers were calculated using standard curves.

### Leaf disc assay and selection of T1 seeds for herbicide resistance

To assess the level of herbicide resistance in primary transgenic plants, leaf disc assays were performed. Several leaf discs cut from the leaves of different transgenic and control plants were placed on regeneration medium in 0.65% agar, containing different concentrations of glyphosate: 0 mM, 0.1 mM and 0.25 mM. Leaf discs were incubated at 26 °C and 16/8 h light/dark cycle for 7 days before taking pictures. Several transgenic and control plants were transferred to soil and grown in the greenhouse to set seeds. The seeds were collected and surface sterilized and germinated on the MS medium with either 100mg/L kanamycin or 0.1 mM glyphosate. The pictures of the seedlings growing on glyphosate were taken 6 weeks after placing the seeds on germination media.

### Tobacco transformation using *EPSPS* as a selectable marker gene

To find out the possibility of using the *CaEPSPS* as a selectable marker gene in plant transformation, tobacco was transformed with a gene construct consisting of the *CaMV35S* promoter driving the expression of the mutated *CaEPSPS* cDNA and with the empty vector, binary plasmid *pCAMBIA 2300*. The transformants were selected with either 100mg/L kanamycin or 0.05 mM glyphosate.

## Results

### *EPSPS* gene from chile shows similarity in nucleotide sequence with *EPSPS* genes from other solanaceous plants

**Fig 1:**
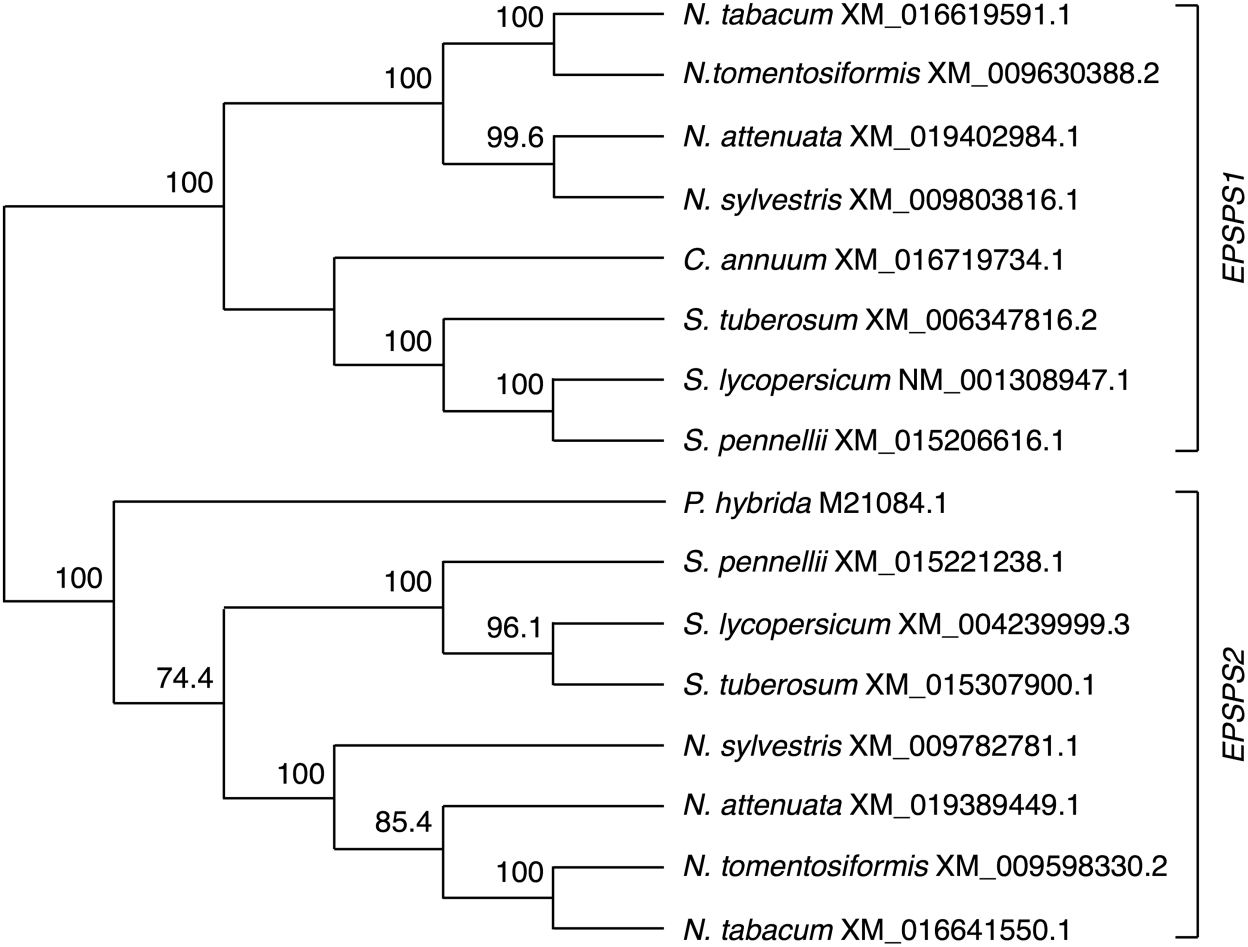
Nucleotide sequence alignment tree of *EPSPS* from plants in the Solanaceae family. *EPSPS* cDNA sequences from *Capsicum annuum*, *Nicotiana attenuata*, *Nicotiana sylvestris*, *Nicotiana tabcum*, *Nicotiana tomentosiformis*, *Petunia hybrida*, *Solanum lycopersicum*, *Solanum pennellii* and *Solanum tuberosum* were subjected to multiple alignment. The Neighbor-joining consensus tree clustered the *EPSPS* sequences in two groups *EPSPS1* and *EPSPS2*. The numbers on the branches represent the percent bootstrap support for 1000 replicates. The tree was rooted with *EPSPS* sequences from plants in the Asterids clade (shown at https://doi.org/10.6084/m9.figshare.5995309.v1). NCBI accession nos. for the solanaceous *EPSPS* nucleotide sequences are indicated.

A full length *EPSPS* cDNA of 1920 bp, including the 5’UTR and the 3’UTR was isolated from the leaves of *Capsicum annuum* cv. NM-64. Sequence comparison of the *Capsicum annum EPSPS* cDNA (*CaEPSPS*) to the *Capsicum* genome assemblies [33, 34] showed that there is only one functional gene encoding *EPSPS* in *Capsicum*, and also showed a high conservation of the *EPSPS* nucleotide sequence among different cultivars, even in the 5’ and 3’ untranslated regions (94% and 98% identity, respectively). Nucleotide sequences of *EPSPS* genes from the Solanaceae family were retrieved from NCBI website to compare with the *Capsicum EPSPS*. Nucleotide sequence alignments clustered the *EPSPS* genes in two homology groups, *EPSPS1 and EPSPS2* (Fig 1). Genes from both groups are found in most solanaceous plants with the exceptions of *Capsicum annuum*, with one gene from group 1 and *Petunia hybrida*, with a gene from group 2. *CaEPSPS* nucleotide sequence showed more than 80% identity to the solanaceous *EPSPS1* class, and 70–80% identity to the *EPSPS2* class.

**Fig 2:**
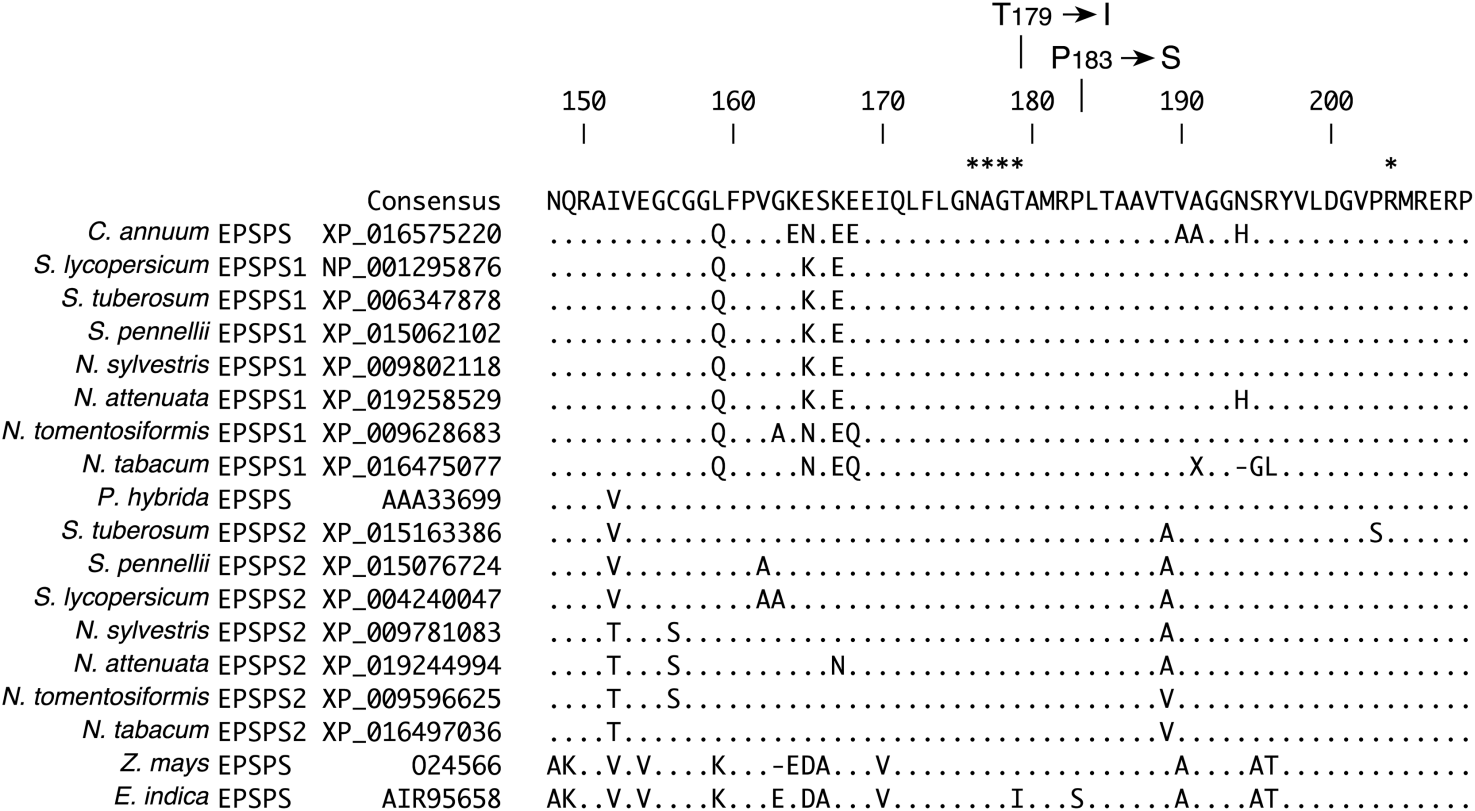
Sequence alignment of the EPSPS proteins from the Solanaceae family. EPSPS amino acid sequences from *Capsicum annuum*, *Nicotiana attenuata*, *Nicotiana sylvestris*, *Nicotiana tabcum*, *Nicotiana tomentosiformis*, *Petunia hybrida*, *Solanum lycopersicum*, *Solanum pennellii* and *Solanum tuberosum* were compared to the sequences of *Zea mays* EPSPS and the EPSPS mutant from *Eleusine indica* by multiple protein alignment. NCBI accession nos. for the protein sequences are indicated. Sequence numbers correspond to their position in the *Capsicum annuum* EPSPS. Only a partial view of the alignment that corresponds to the substrate binding region is shown. Dots represent conserved amino acids and dashes represent deletions. Amino acid residues involved in binding of the phospho*enol*pyruvate are marked with asterisks [10]. The amino acids 179 and 183, replaced by site-directed mutagenesis are indicated.

All the solanaceous *EPSPS* sequences retrieved encode plastidic proteins as the presence of a transit peptide predicts. Amino acid sequence alignments showed high conservation of the EPSPS among the Solanaceae family, with more than 90% identity in the sequence of the mature protein. The sequences of the EPSPS transit peptide, however, are more divergent with 50–75% identity among the Solanaceae. Protein sequence alignments also indicate a strong conservation of the solanaceous EPSPS proteins in their active site (Fig 2). The EPSPS from *Zea mays* and *Eleusine indica* show more sequence divergence from the solanaceous EPSPS proteins.

### Overexpression of the wild type *EPSPS* gene from chile did not confer resistance to glyphosate in tobacco

**Fig 3:**
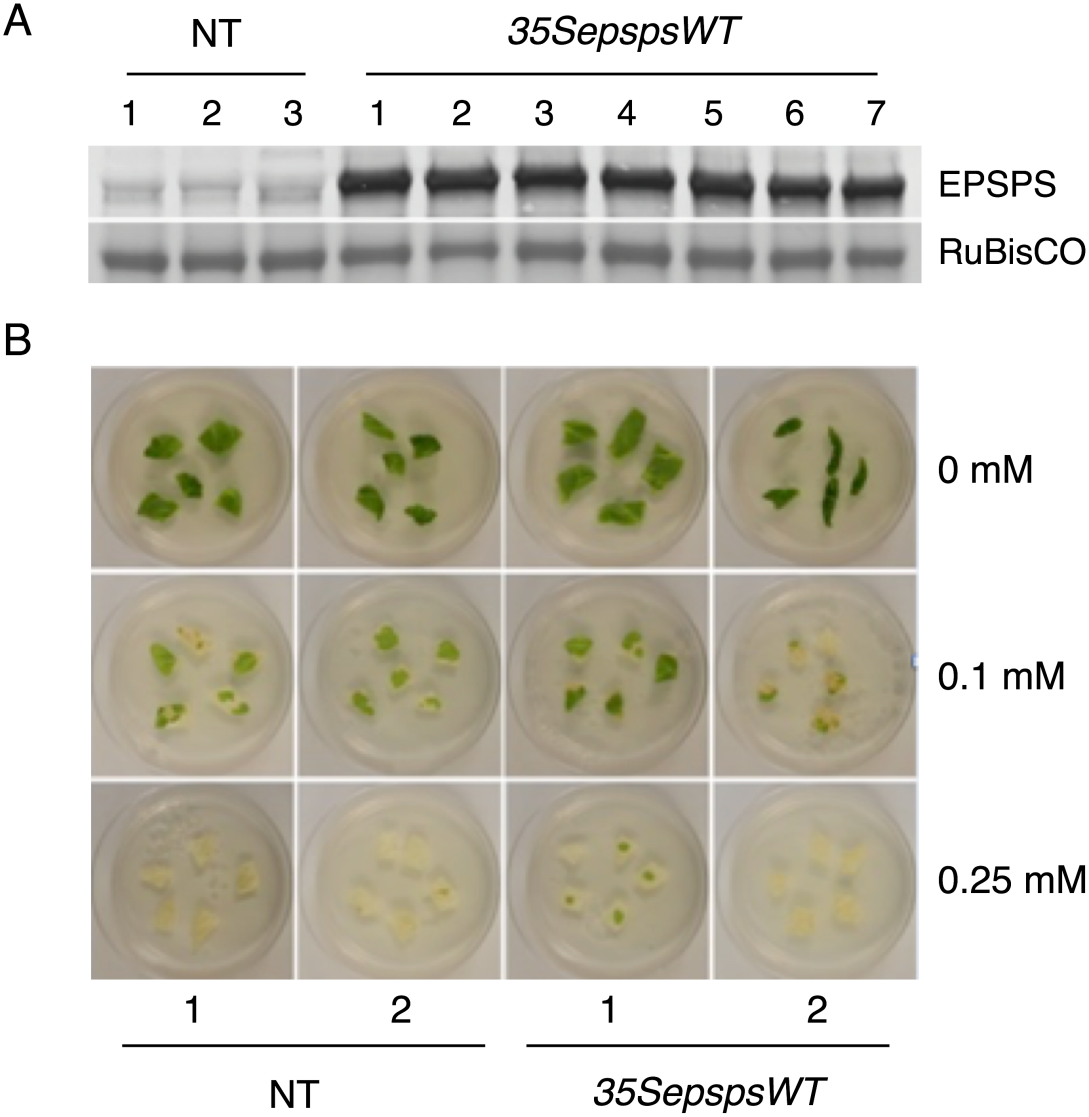
Testing the efficacy of the *CaEPSPS* wild type gene in conferring tolerance to glyphosate in transgenic tobacco. (A) Western blot analysis of control plants (NT) and *CaMV35S*::*CaEPSPS* transformants (*35SepspsWT*). Protein extracted from the leaves was subjected to SDS PAGE followed by western blotting using antibodies raised to chile EPSPS protein. (B). Leaf discs from non-transformed control plants (NT) and *35SepspsWT* transformants were placed on the regeneration medium in 0.65% agar containing glyphosate at concentrations of 0 mM, 0.1 mM and 0.25 mM). Pictures were taken after 7 days on glyphosate media.

Glyphosate binds to EPSPS protein at the active site and inhibits the catalytic activity of the enzyme, thus causing the plant to die because of a shortage of aromatic amino acids. However, if EPSPS levels could be increased in plants, the EPSPS in excess of what is needed for the synthesis of aromatic amino acids could titrate out the glyphosate that is applied and allow the plants to become glyphosate tolerant. Based on this rationale, we transformed tobacco plants with the unmodified *CaEPSPS* cDNA driven by the *CaMV35S* promoter (*35SepspsWT*). The transformants were identified based on their ability to grow on media containing Kanamycin (100 mg/L) and were confirmed positive by performing genomic PCR. Protein extracted from leaf tissues from several putative *35SepspsWT* transformants and control plants were subjected to western blot analysis using EPSPS antibodies. As seen in Fig 3, the results of two representative plants show that both control and *CaMV35SepspsWT* transformants showed an immunoreactive band of ~50 kD. However, the steady state level of the immunoreactive band in the *35SepspsWT* transformants was 5 to 7-fold higher than the control plants as determined by the quantification of the immunoreactive bands.

To check if the increased level of EPSPS protein would confer resistance to glyphosate, leaf pieces from *35SepspsWT* transformants and NT plants were placed on different concentrations of glyphosate; 0 mM, 0.1 mM, 0.25 mM. As seen in Fig 3, after 7 days of incubation, the leaf pieces from the *35SepspsWT* transformants showed minimal protection against glyphosate even at the low concentration of 0.1 mM, like the NT plants.

### A mutated form of *EPSPS* conferred resistance to glyphosate in tobacco

**Fig 4:**
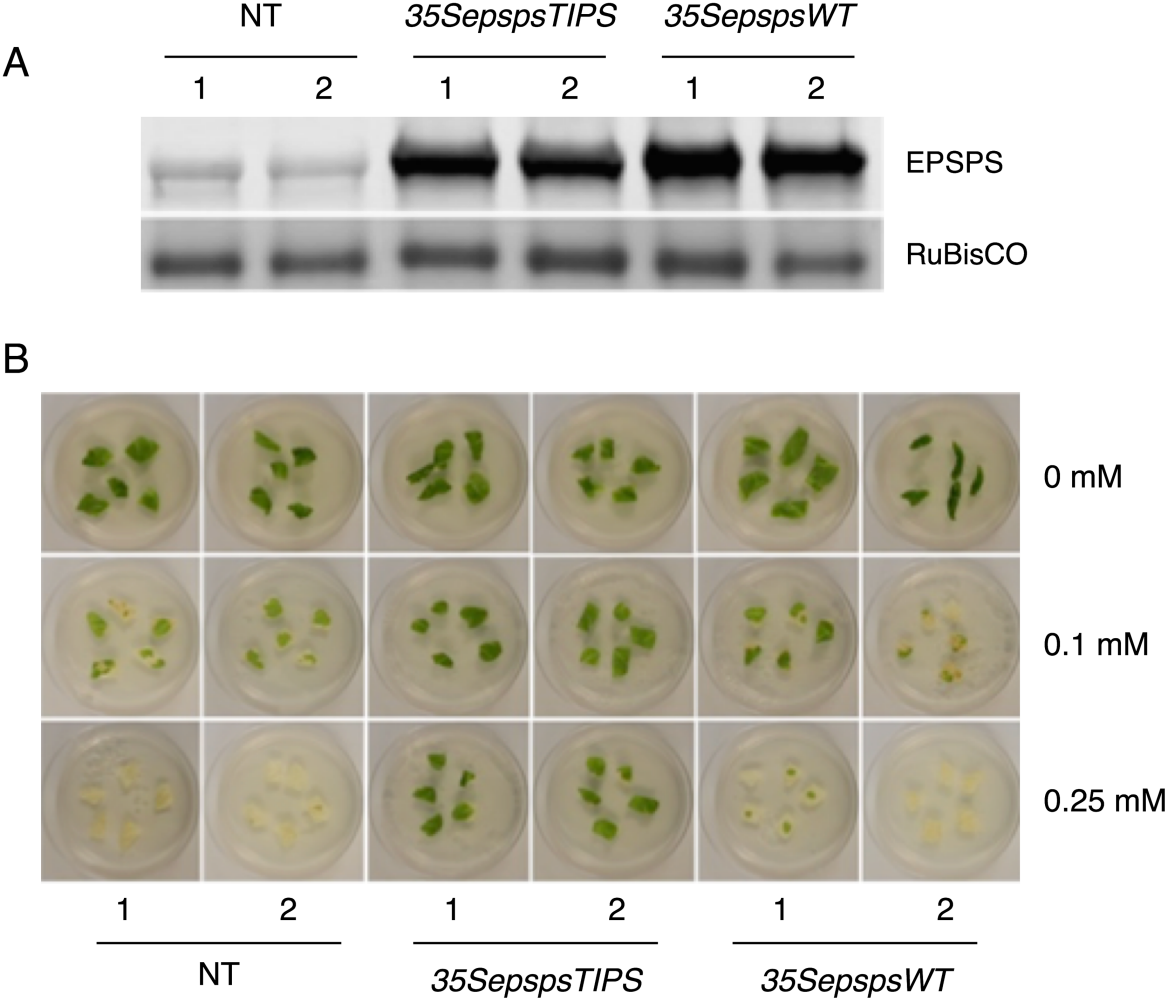
Comparing the effectivity of mutated *CaEPSPS* with the WT *CaEPSPS* in conferring tolerance to glyphosate in transgenic tobacco. (A). Western blot analysis of control plants (NT) and plants transformed with the mutated *CaEPSPS* (*35SepspsTIPS*) and the unmodified *CaEPSPS* (*35SepspsWT*) genes driven by the *CaMV35S* promoter. Protein extracted from the leaves was subjected to SDS PAGE followed by western blotting using antibodies raised to chile EPSPS protein. (B). Leaf discs from non-transformed control plants (NT), mutated *CaEPSPS* (*35SepspsTIPS*) and unmodified *CaEPSPS* (*35SepspsWT*) transformants were placed on the regeneration medium in 0.65% agar containing glyphosate at concentrations of 0 mM, 0.1 mM and 0.25 mM. Pictures were taken after 7 days on glyphosate media.

The results from our previous experiment suggested that over-expression of the WT *CaEPSPS* gene resulting in a 7-fold increase in EPSPS level, did not confer resistance to glyphosate in tobacco plants. For the next step, we introduced mutations in the *CaEPSPS* gene to change Thr179 to Ile and Pro183 to Ser (TIPS, Fig 2). The mutated gene construct driven by the *CaMV35S* promoter (*35SepspsTIPS*) was introduced into tobacco and several independent transformants were obtained. We selected two transformants for further analysis. Protein extracts from the leaves of the two classes of transformants along with the control plants were subjected to western blot analysis using the chile EPSPS antibodies. The level of EPSPS accumulation in *35SepspsTIPS* transformants was less than in the *35SepspsWT* transformants, suggesting that the modified protein may not be as stable as the unmodified (WT) EPSPS protein (Fig 4).

To check if the mutated gene product is more effective in glyphosate tolerance, we performed cut leaf assay by incubating leaf material from the two classes of transformants and NT plants on media containing 0.1 and 0.25 mM glyphosate. As seen in Fig 4, the leaf discs from the control plants and *35SepspsWT* plants showed extensive bleaching in 0.1 mM glyphosate and were completely bleached out in 0.25 mM glyphosate. In contrast, the *35SepspsTIPS* transformants showed 100% resistance in 0.1 mM and > 90% protection in 0.25 mM glyphosate suggesting that the EPSPS mutant protein was more resistant to glyphosate than the WT protein.

### Tobacco transformants with the chile *EPSPS* promoter driving the mutated *CaEPSPS* gene showed limited resistance to glyphosate

**Fig 5:**
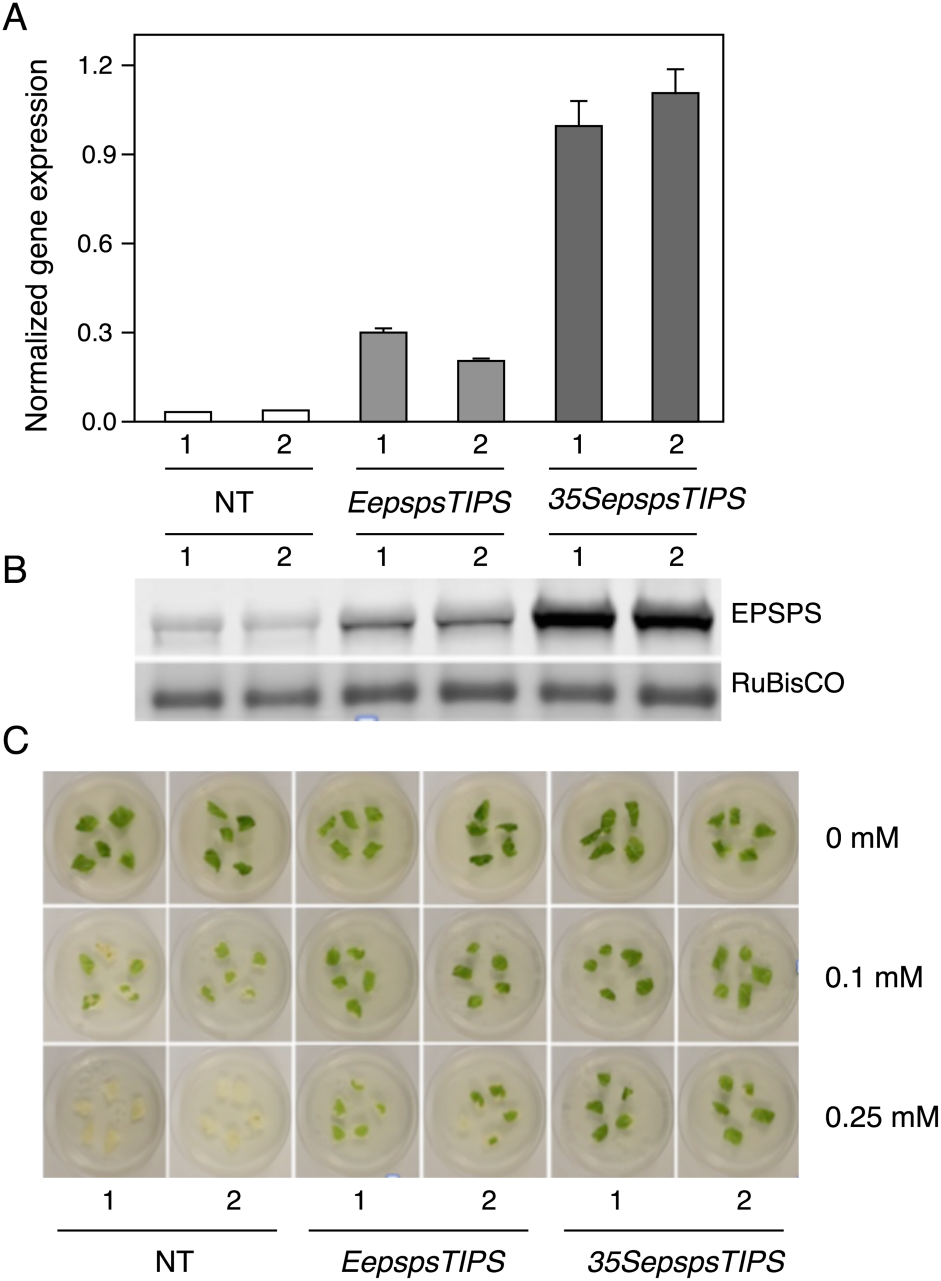
Comparison of the chile *EPSPS* promoter driving the mutated *CaEPSPS* with the *CaMV35S* promoter driving the mutated *CaEPSPS* in conferring tolerance to glyphosate in transgenic tobacco. (A) qRT-PCR analysis: RNA isolated from plants containing the *CaMV35S* driving the mutated *EPSPS* cDNA (*35SepspsTIPS*) or the *EPSPS* promoter driving the mutated *CaEPSPS* cDNA (*EepspsTIPS*) along with NT plants, were subjected to qRT-PCR using *CaEPSPS*-specific primer sets and normalized against *EF-1α* as the internal reference gene. B) Western blot analysis of control non-transformed plants (NT) and plants transformed with the mutated *CaEPSPS* cDNA driven by the *CaEPSPS* promoter (*EepspsTIPS*) or the *CaMV35S* promoter (*35SepspsTIPS*). Protein extracted from the leaves was subjected to SDS PAGE followed by western blotting using antibodies raised to chile EPSPS protein. (C) Leaf discs from non-transformed control plants (NT), and from plants transformed with the *EepspsTIPS and 35SepspsTIPS* gene constructs were placed on the regeneration media in 0.65% agar containing glyphosate at concentrations of 0 mM, 0.1 mM and 0.25 mM. Pictures were taken after 7 days on glyphosate media.

With the goal to develop a cisgenic/intragenic method to make chile plants resistant to glyphosate, a gene construct consisting of the native chile *EPSPS* gene promoter driving the mutated *CaEPSPS* gene (*EepspsTIPS*) was introduced into tobacco. To compare the *EPSPS* transcript level in the plants transformed with the *CaMV35S* driven mutated gene (*35SepspsTIPS*) or the *EPSPS* promoter driving the mutated gene (*EepspsTIPS*), total RNA was isolated from the leaves of the transformants, along with NT plants, and subjected to qRT-PCR using *CaEPSPS*-specific primer sets. Tobacco EF-1α was used as the internal reference gene[32]. The *EPSPS* transcript level in the *EepspsTIPS* transformants was ~3 to 4 fold lower than in the *35SepspsTIPS* transformants (Fig 5). The leaf proteins from the same plants as used for RNA isolation were subjected to western blot analysis using the chile EPSPS antibodies. The EPSPS protein in the leaves of the two classes of transformants followed the same trend as the transcript level, the level in the *EepspsTIPS* plants being about 3 to 4 fold lower than in the *35SepspsTIPS* transformants (Fig 5). These results would suggest that the chile *EPSPS* promoter is functionally weaker than the *CaMV35S* promoter in the leaves of tobacco plants.

The leaves of the two classes of transformants (*EepspsTIPS and 35SepspsTIPS*) along with control plants were subjected to the cut leaf assay. The *EepspsTIPS* transformants showed tolerance to glyphosate but to a smaller extent compared to the *35SepspsTIPS* transformants (Fig 5). However, the leaves of the *EepspsTIPS* transformants showed a higher level of resistance to glyphosate compared to the *35SepspsWT* (Fig 4) even though the *EepspsTIPS* transformants have a lower level of EPSPS when compared to the level in *35SepspsWT* transformants.

### Glyphosate resistance can be enhanced by increasing the number of copies of the *CaEPSPS* transgene

**Fig 6:**
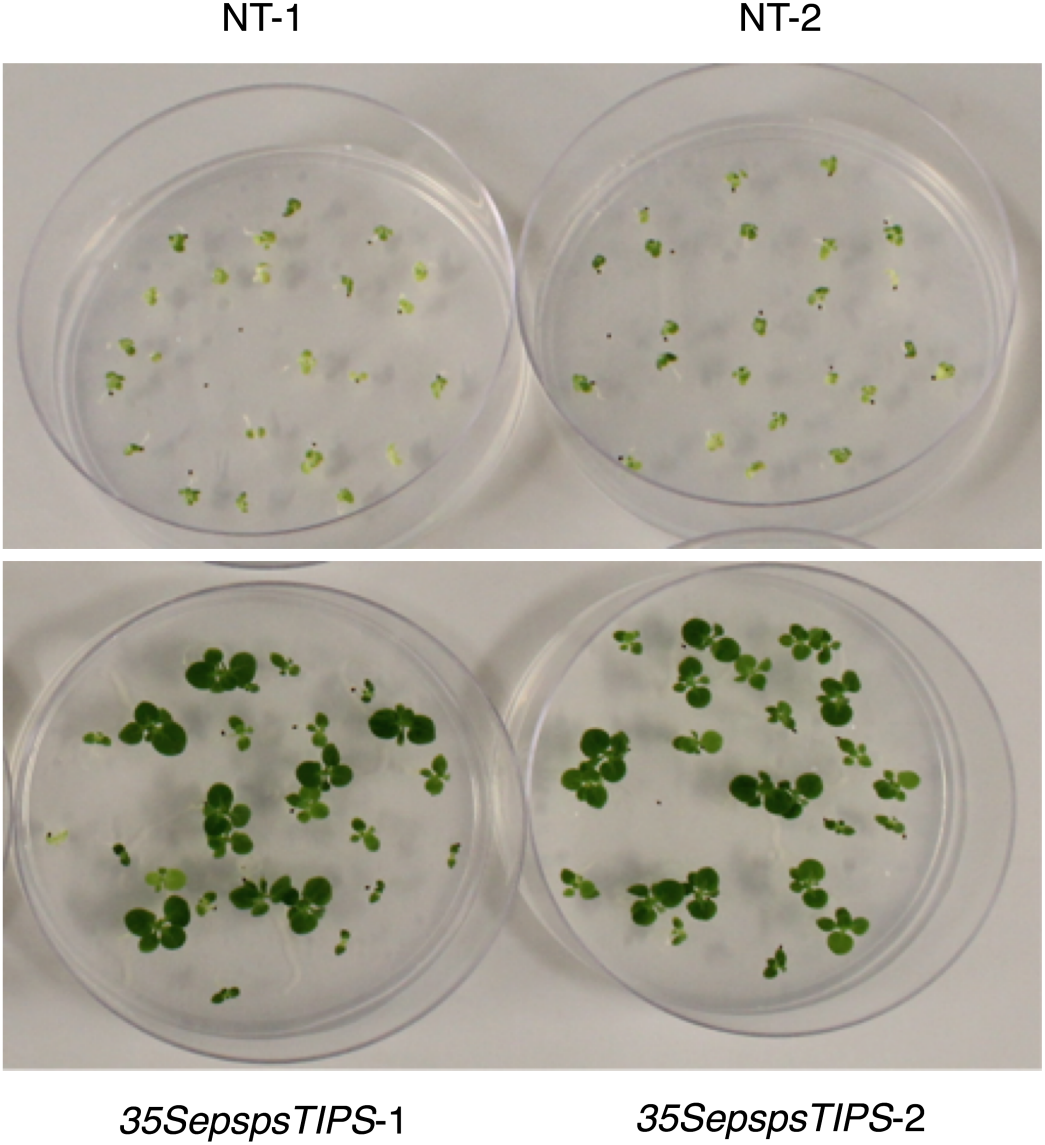
Selection of T1 progeny of nontransformed plants and the mutated *CaEPSPS* transformants on glyphosate. Seeds obtained by selfing non transformed plants (NT) and the primary transformants harboring the mutated *CaEPSPS* driven by the *CaMV35S* promoter (*35SepspsTIPS*) were germinated on agar containing 0.1 mM glyphosate. Pictures were taken 6 weeks following placement of seeds on agar.

We rationalized that transformants homozygous for the transgene would be more resistant to glyphosate compared to the hemizygous primary transformants, by virtue of having more copies of the transgene. The seeds produced by selfing the primary transformants were selected on either kanamycin (100 mg/L) or glyphosate (0.1mM). All the three classes of transformants germinated on kanamycin and in each case, the categorization of seedlings based on size, typified the Mendelian genotypic ratio of 1:2:1 (28:64:23). The NT seeds germinated but the seedlings did not grow any further. When the same seeds were germinated on media containing 0.1 mM glyphosate (Fig 6), the seeds from the *EepspsTIPS* and the *35SepspsWT* transformants germinated but the seedlings showed minimal growth. The *35SepspsTIPS* transformants, however, showed seedlings of three sizes, following the 1:2:1 Mendelian ratio (Fig 6). We would conclude that the larger seedlings have 2 alleles for the transgene, the next set of seedlings have 1 allele each, while the ones that show minimal growth are the ones that do not have an allele for the glyphosate-resistant gene. This set of experiments suggests that resistance to glyphosate could be improved by increasing the number of the mutated *EPSPS* genes in the genome of the transformants.

### Mutated *CaEPSPS* could be used as a selectable marker in transformation

**Fig 7:**
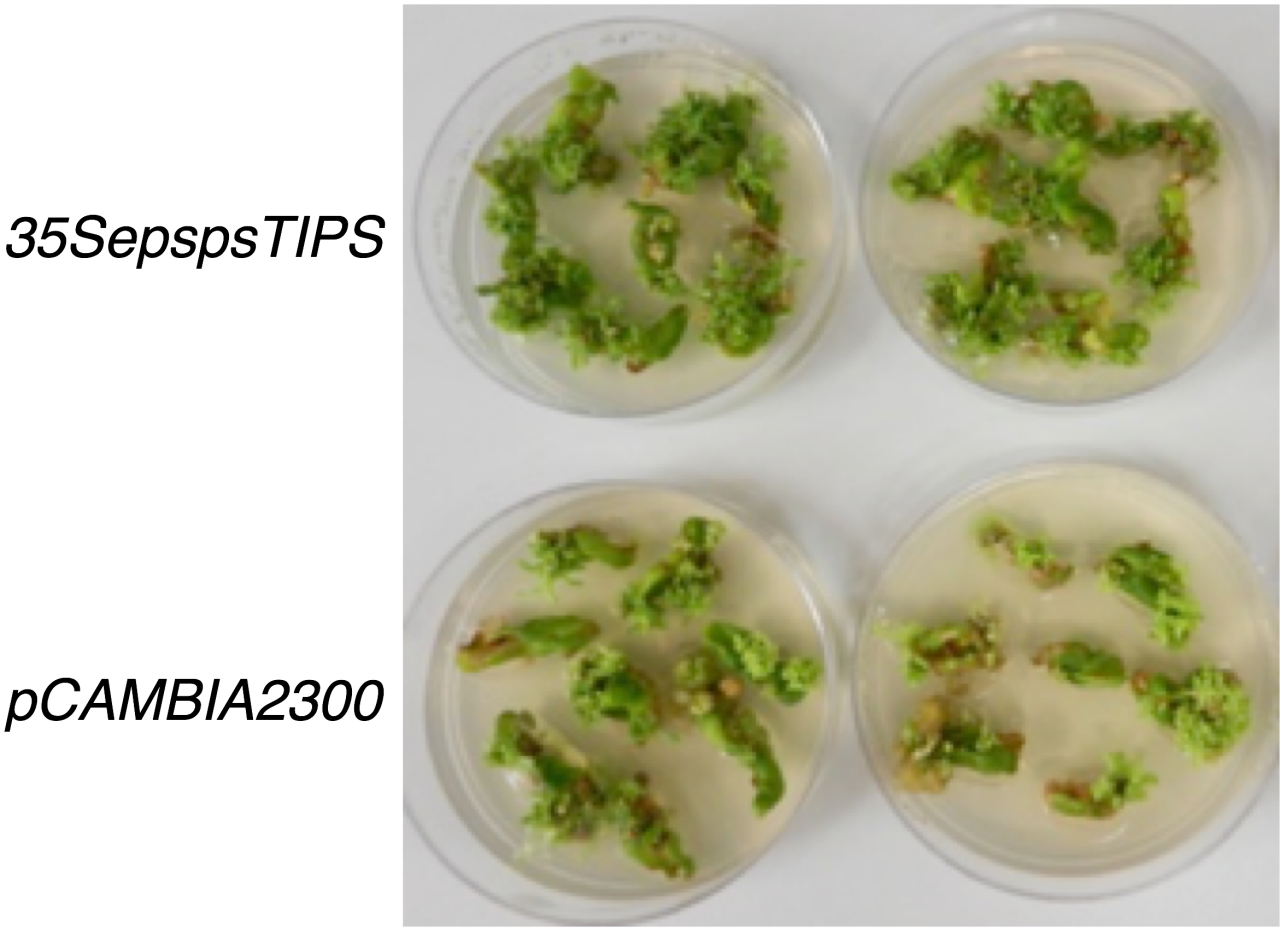
Transformation of tobacco with *Agrobacterium tumefaciens* with an empty vector (*pCAMBIA2300*) and the gene construct containing the *mutated CaEPSPS* driven by the *CaMV35S* promoter (*35SepspsTIPS*). Tobacco leaf discs were transformed using the protocol described in ‘Materials and Methods’ and the cultures were placed on selective media containing 0.05 mM glyphosate. The picture was taken 8 weeks following transformation.

One of the points to consider while developing a cisgenic/intragenic approach is also to use a selectable marker that is indigenous to the plant being engineered. To check if the *EPSPS* transgene could be used to select transformed plants, tobacco leaves were transformed with the mutated *CaEPSPS* gene constructs *EepspsTIPS* and *35SepspsTIPS*, and the empty vector *pCAMBIA 2300*, using 0.05 mM glyphosate for selection. After 6 weeks of culturing, the *EepspsTIPS* like the plants transformed with the empty vector, showed very little callusing and extensive bleaching. As such, we used 35SepspsTIPS to transform tobacco along with the *pCAMBIA2300* as control. After 8 weeks of culturing on 0.05 mM glyphosate media, the leaf discs transformed with the *35SepspsTIPS* showed good proliferation of green calli with small embryo like structures, while the empty vector transformed leaf discs did not callus as well and showed some degree of bleaching (Fig 7). We concluded that while resistance to glyphosate could potentially be used for selection of transformants, the glyphosate-resistant EPSPS levels in the *35SepspsTIPS* transformed leaves was not high enough for the development of embryos into plantlets in the presence of glyphosate even though the concentration of glyphosate was very low. For a cisgenic/intragenic approach, the appropriate gene construct should be *EepspsTIPS*, however, the weak promoter strength of the chile *EPSPS* gene would preclude its use as a selectable marker. For a more robust selection system, we need a chile gene promoter that is stronger than the *EPSPS* promoter and an EPSPS protein much more resistant to glyphosate (i.e. mutations of other residues).

### Chile transformants with the mutated *EPSPS* gene construct showed an increase in resistance to glyphosate

**Fig 8:**
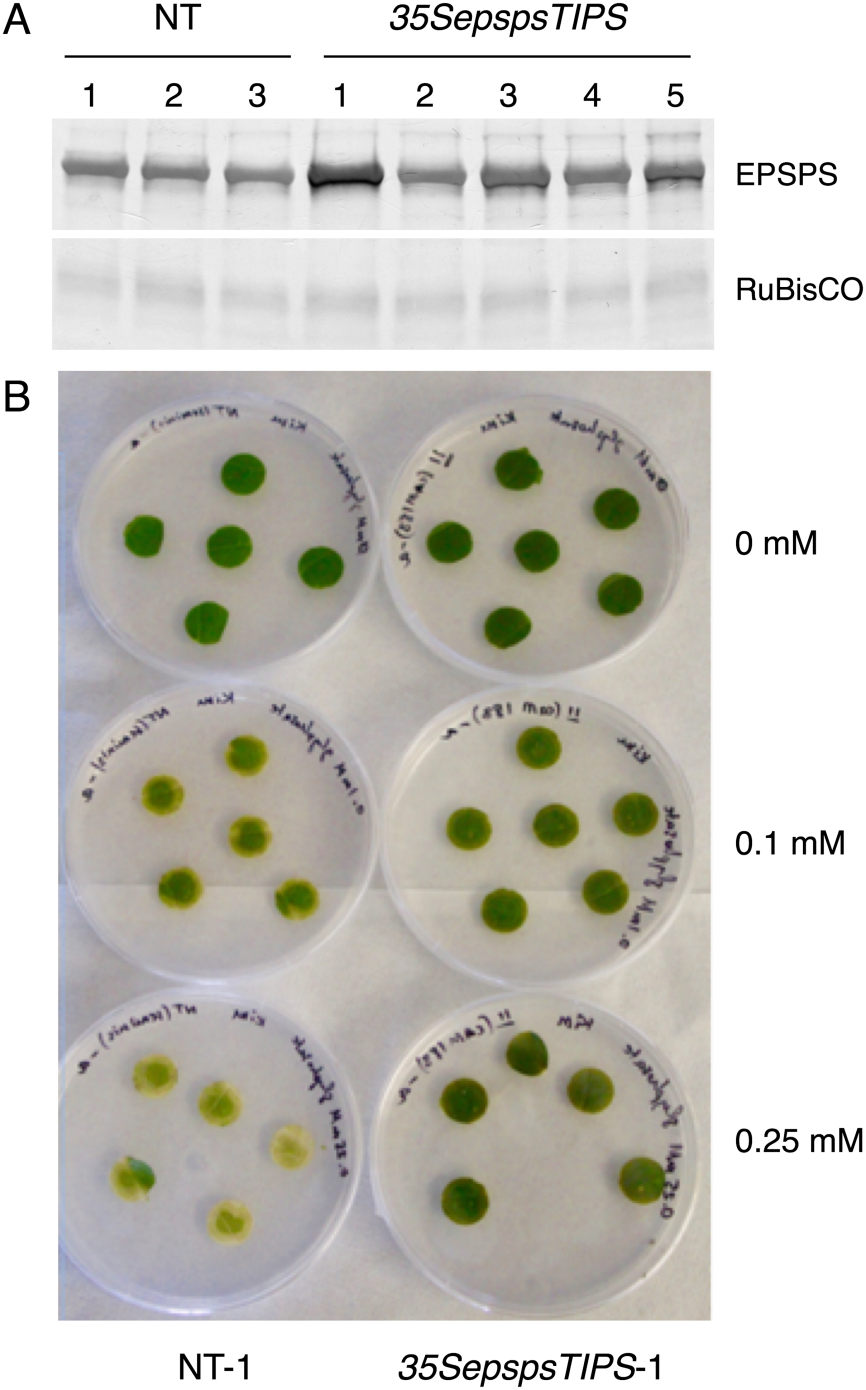
Checking the efficacy of the mutated *CaEPSPS* gene in conferring tolerance to glyphosate in transgenic chile. (A). Western blot analysis of control non-transformed plants (NT) and plants transformed with the *CaMV35S* promoter driving the mutated *CaEPSPS* cDNA (*35SepspsTIPS*). Protein extracted from the leaves of control and transformed chile plants were subjected to SDS PAGE followed by western blotting using antibodies raised against chile EPSPS protein. The signal for the RuBisCO small subunit is shown as a loading control. (B). Leaf discs from non-transformed control plant NT-1 and *35SepspsTIPS*-1 transformants were placed on the regeneration medium in 0.65% agar containing glyphosate at concentrations of 0 mM, 0.1 mM and 0.25 mM. Pictures were taken after 7 days on glyphosate media.

Since the only gene construct that conferred some degree of resistance to glyphosate in tobacco was the mutated *CaEPSPS* driven by the *CaMV35S* promoter (*35SepspsTIPS*), we transformed chile with this gene construct. Protein extracted from the leaves of some of the putative chile transformants was subjected to western blot analysis and as seen in Fig 8, an immunoreactive band of ~50 kD was seen in all the lanes including the control plants. It is interesting to point out that the EPSPS level in chile leaves was higher than in tobacco leaves but it is also possible that the antibodies have higher affinity for chile EPSPS. While all the transformants showed increase in the EPSPS level over control, transformants *35SepspsTIPS*-1 and *35SepspsTIPS*-3 showed a sizeable increase in the level of EPSPS protein.

Leaf discs from both the *35SepspsTIPS*-1 and *35SepspsTIPS*-3 chile transformants were subjected to the cut leaf assay. Both the transformants showed some resistance to glyphosate. The data from the assay using *35SepspsTIPS*-1 leaf discs is shown in Fig 8. While the NT plants started exhibiting loss of chlorophyll in 0.1mM concentration of glyphosate, they were completely bleached on media with 0.25 mM glyphosate, the leaf discs from the *35SepspsTIPS* transformants exhibited little bleaching even at a concentration of 0.25 mM. The results suggest that introducing a chile gene into chile does not cause co-suppression and the mutated *EPSPS* gene driven by the *CaMV35S* promoter does confer resistance to glyphosate. In fact the tolerance to glyphosate in chile seems higher than in the tobacco transformants (Fig 5).

## Discussion

There have been many reports on the production of glyphosate resistant crops like rice, wheat, maize and soybean using either glyphosate resistant *EPSPS* genes from bacteria, genetically engineered plant *EPSPS* genes and synthetic genes [18, 20, 35]. Tobacco, petunia and Arabidopsis have also been used to check the efficacy of different *EPSPS* genes. However, no attempts have been made to use transgenic approaches to introduce important traits like glyphosate resistance in chile (*Capsicum annuum*), which is an important plant of great economic importance all over the world. One of the impediments in taking a transgenic approach in chile for any trait has been two-fold: i. transformation of chile is not routine and transformation efficiency is low; ii. chile growers are opposed to introducing foreign DNA, into chile plants.

The objective of this project was to use an intragenic approach to develop chile plants that are resistant to glyphosate. Towards developing the intragenic approach for glyphosate resistance in chile, we used tobacco as the initial plant to test the different gene constructs with the rationale that tobacco is a solanaceous plant and tobacco transformation is routine. Overexpression of *CaEPSPS* resulting in a 5 to 7 fold increase in EPSPS levels in the tobacco transformants, exhibited little to no resistance to even very low concentration of glyphosate. Earlier studies [36] had shown that petunia plants transformed with an *EPSPS* gene isolated from a glyphosate-tolerant petunia hybrid cell line and driven by the *CaMV35S* promoter, were glyphosate tolerant. It is difficult to explain why the EPSPS protein from petunia and chile which share ~90% sequence similarity show differences in glyphosate-resistance but we cannot rule out the possibility that the EPSPS protein from this petunia cell line was resistant to glyphosate, notwithstanding that this line was deemed resistant due to *EPSPS* gene amplification (36). More recently, it has been shown that Arabidopsis plants transformed with an Arabidopsis *EPSPS* gene driven by the *CaMV35S* promoter was tolerant to glyphosate [37]. In another study, it was shown that a an *EPSPS* gene from *Convolvulus arvensis* driven by the *CaMV35S* promoter also conferred resistance to glyphosate in Arabidopsis [38]. The Arabidopsis transformants in either study, were not checked for EPSPS protein levels relative to the control plants thus making it difficult to compare the outcome of the present study with those previous studies [37,38]. The plant background, tobacco versus Arabidopsis, could also contribute to the differences in the results. Moreover, there could be differences between the chile EPSPS protein with that of Arabidopsis and *Convolvulus* EPSPS protein, even though the critical residues in the catalytic region of all the EPSPS proteins analyzed are conserved [29]. While the tobacco transformants used in this study were the primary transformants (hemizygous), the Arabidopsis plants used in the the other two studies were homozygous for the transgene.

One of the more successful strategies for developing glyphosate resistance in plants has involved transforming plants with genes encoding for glyphosate resistant EPSPS protein. These glyphosate resistant EPSPS proteins, while being catalytically active cannot bind to glyphosate. There are many reports of such enzymes that have been produced by *in vitro* methods [7, 13, 18, 39]. The mutations that have been shown to be most effective are the simultaneous substitutions at positions 102 (threonine by isoleucine) and 106 (proline by serine) (TIPS) [4, 18]. We introduced these mutations into the chile *EPSPS* gene. The tobacco transformants with the TIPS mutated *CaEPSPS* gene showed resistance to glyphosate but not at levels seen in maize with the mutated corn TIPS EPSPS protein. The highly glyphosate-resistant EPSPS protein in *E*. *indica*, also has the same substitutions (TIPS) as the chile gene in this study [23]. Based on the various reports of glyphosate-resistant EPSPS, it appears that maize takes the central position both with regards to the gene (TIPS) and the plant [18]. In petunia, the glyphosate resistant EPSPS was produced by mutations other than TIPS [18]. Thus the TIPS mutation that is so effective in maize EPSPS and which is naturally present in the *E*. *indica* EPSPS protein may not be as effective in the EPSPS protein from solanaceous plants which would include the chile EPSPS protein. This could be attributed to some minor but important differences in the amino acid sequences inside and outside the catalytic region. The amino acid sequence in the active site of the solanaceous EPSPS protein show significant differences with those of *Z*. *mays* and *E*. *indica*, supporting the idea that the TIPS mutation is not as effective in EPSPS from *C*. *annuum* as compared to *Z*. *mays* and *E*. *indica* (Fig 2).

Tobacco seedlings homozygous for the mutated *CaEPSPS* gene in this study showed higher level of resistance to glyphosate compared to the tobacco seedlings hemizygous for the transgene (Fig 6), suggesting that increasing the copy number of the *CaEPSPS* transgene could be one way to increase the ability of the transformed plants to tolerate glyphosate. One could envision that the introduction of multiple copies of the mutated gene, could allow for the production of enough of the glyphosate tolerant enzyme, to make the plant resistant to glyphosate. Gene amplification of the *EPSPS* gene is what confers glyphosate resistance in some glyphosate resistant weeds [40, 41]. It is a feasible approach to introduce multiple copies of the gene as a tandem repeat, but there is always the likelihood of co-suppression [42]. Moreover, the plants may not be able to handle a huge increase in the EPSPS protein because this could deregulate the whole shikimate pathway and affect other metabolic pathways, which may explain the reduced growth of the *E*. *indica* plants homozygous for the TIPS mutation [23].

Towards developing an intragenic approach to confer glyphosate resistance in chile, we isolated the chile *EPSPS* promoter and engineered it in front of the mutated *CaEPSPS* gene (*EepspsTIPS*). Since there is only one functional *EPSPS* gene in chile [33, 34], we assume that the *EPSPS* promoter that we isolated was functional. Based on the level of accumulation of the transgene product in the leaves, it would appear that the *EPSPS* promoter is weaker than the *CaMV35S* promoter in the leaves of tobacco (Fig 5). The transformants with the *EPSPS* promoter driving the mutated *CaEPSPS* gene, however, showed higher level of resistance to glyphosate when compared to the *CaMV35S* driving the WT gene in the leaf disc assay (Fig 5). However, a promoter that is stronger than the *EPSPS* promoter has to be used. The RuBisCO small subunit promoter could be one such promoter. However, use of this promoter will give protection only to the leaves, but that may be enough since glyphosate is usually used as an aerial spray.

For an intragenic approach, the glyphosate tolerant *CaEPSPS* gene could also serve as a selectable marker in plant transformation. We tested the possibility of using glyphosate for selecting transformed tobacco cells and as seen in Fig 5, the transformed cells with *35SepspsTIPS* were greener and had more green plantlets compared to the cells transformed with the empty vector. However, the plantlets did not grow into plants. This is most likely due to the fact that the mutated EPSPS enzyme was not effective enough in resisting even the low level of glyphosate included in the media. However, since there was a distinct difference between the *35SepspsTIPS* and the plants transformed with the empty vector, with regards to cell proliferation and emergence of embryos, we would infer that the mutated *CaEPSPS* could function as a selectable marker. A number of *EPSPS* genes of plant and bacterial origin have been used as a plant selectable marker in soybean and corn [43, 44].

While our objective was to use an intragenic approach to produce chile plants that are resistant to glyphosate, we used tobacco to identify the most effective gene construct that could confer resistance to glyphosate. We introduced the gene construct *35SepspsTIPS* into chile using the transformation protocol that we have developed/modified in our lab. The chile transformants with the *35SepspsTIPS* construct showed higher resistance to glyphosate compared to tobacco transformants with the same construct. An important point to make is that the chile transgene in chile did not result in co-suppression. While the modified EPSPS protein did confer reasonable level of resistance to glyphosate in the primary chile transformants, we would expect plants that are homozygous for the transgene to exhibit higher level of resistance. Now that we have shown that an intragenic approach can be used to confer glyphosate-resistance, future studies will focus on obtaining a stronger promoter, like the Rubisco small subunit promoter and an *EPSPS* gene that is more resistant to glyphosate, by introducing substitutions similar to those found in other glyphosate-resistant bacterial EPSPS proteins [39, 45, 46].

While our long term goal is to produce glyphosate resistant chile plants using an intragenic approach, the focus of this study was to test the feasibility of this approach. Towards this objective, we have used tissue culture grown plants to conduct a glyphosate tolerance test using leaf discs placed on agar plates containing the different concentrations of glyphosate. In the field, on the other hand, fully grown hardened plants growing along with weeds are sprayed with glyphosate. The tissue culture approach involves a continuous exposure to the herbicide for long periods of time while application of the herbicide as a spray is rather transient. Thus it is difficult to say whether the mature transformants (*35SepspsTIPS*), if grown in the field, could handle the concentrations of glyphosate used by farmers to kill weeds. Our study, however, can say with certainty that the concentrations of glyphosate that we used in our experiments were very effective in killing control plants but not the transformants (Fig 3). The control plants used in our leaf disc assay along with the *35SepspsTIPS* chile transformants, would be comparable to the weeds growing along side the transformed plants in the field.
